# Supplementary material for: Progression of Non-Significant Mitral and Tricuspid Regurgitation after Surgical Aortic Valve Replacement for Aortic Regurgitation
Source: J Clin Med. 2023 Sep 29;12(19):6280. doi: 10.3390/jcm12196280 (PMC10573116; doi:10.3390/jcm12196280)
Supplement: Supplementary file 1 [file jcm-12-06280-s001.zip › jcm-2593898-supplementary.pdf]

Supplemental Table S1. Morality Along the Entire Follow-Up Period

	**Total Cohort (N=184)**	**Primary Outcome (N=36)**	**No Primary Outcome (N=148)**	**P-Value**
All-Cause	49 (26.6)	19 (52.8)	30 (20.3)	<0.001
Cardiovascular	18 (36.7)	8 (42.1)	10 (33.3)	0.081
Non-Cardiovascular	31 (63.3)	11 (57.9)	20 (66.7)	0.081

Data are presented as number (percent).

Supplemental Table S2. Rates of Moderate or Severe Mitral and/or Tricuspid Regurgitation on the Last Echocardiogram According to Mitral and/or Tricuspid Regurgitation Severity at Baseline

	**Baseline MR Up-to-Mild**	**Baseline MR Mild-to-Moderate**	**P-value**	**Baseline TR Up-to-Mild**	**Baseline TR Mild-to-Moderate**	**P-value**	**Baseline MR/TR Up-to-Mild**	**Baseline MR/TR Mild-to-Moderate**	**P-value**
Last MR Moderate or Severe	12 (8.4)	8 (19.5)	0.083	18 (10.7)	1 (7.1)	1.000	12 (8.8)	8 (17.0)	0.116
Last TR Moderate or Severe	13 (9.1)	12 (29.3)	0.001	18 (10.7)	6 (42.9)	0.004	12 (8.8)	13 (27.7)	0.001
Last MR/TR Moderate or Severe	21 (14.7)	15 (36.6)	0.002	29 (17.2)	6 (42.9)	0.030	20 (14.6)	16 (34.0)	0.004

Data are presented as number (percent).

MR = mitral regurgitation; TR = tricuspid regurgitation

Supplemental Table S3. Univariable Binary Logistic Regression Model for the Primary Outcome

	OR (95% CI)	P-Value
**Baseline Clinical Characteristics**		
Age		
Continuous	1.03 (0.99-1.06)	0.091
≥65 years	1.65 (0.79-3.44)	0.185
Sex Male	0.53 (0.24-1.18)	0.119
Body Surface Area, Mosteller Formula (continuous)	0.28 (0.05-1.56)	0.147
Body Mass Index (kg/m^2)	1.00 (0.99-1.01)	0.970
Obesity	0.56 (0.24-1.34)	0.194
Hypertension	0.96 (0.43-2.13)	0.921
Diabetes Mellitus	1.00 (0.45-2.21)	0.991
Dyslipidemia	1.21 (0.48-3.03)	0.682
Smoking History	0.58 (0.21-1.61)	0.292
Estimated Glomerular Filtration Rate, Cockcroft Formula (mL/kg/min)	1.00 (0.99-1.01)	0.403
Stage ≥III Chronic Kidney Disease	1.41 (0.54-3.70)	0.484
Ischemic Heart Disease	2.00 (0.95-4.21)	0.067
Prior Stroke / Transient Ischemic Attack	2.47 (0.94-6.47)	0.067
Atrial Fibrillation/Flutter	3.33 (1.55-7.13)	0.002
Cardiac Implantable Electronic Device	1.70 (0.60-4.79)	0.316
New York Heart Association Class		
Continuous	1.92 (1.14-3.21)	0.014
≥II	3.30 (1.45-7.52)	<0.001
**Baseline Echocardiographic Parameters**		
Pure Aortic Regurgitation	2.12 (0.82-5.44)	0.120
Acute Aortic Regurgitation	1.35 (0.14-13.42)	0.796
Severe Aortic Regurgitation	1.28 (0.62-2.65)	0.511
Aortic Regurgitation Etiology		
Annular Dilatation	0.90 (0.36-2.27)	0.828
Leaflet Prolapse/Flail	1.62 (0.46-5.67)	0.451
Leaflet Restriction	1.71 (0.41-7.17)	0.460
Endocarditis	0.24 (0.03-1.93)	0.180
Aortic Dissection	3.92 (0.24-64.91)	0.341
Moderate and Above Aortic Stenosis	0.42 (0.16-1.07)	0.068

Mean Aortic Pressure Gradient (continuous)	0.99 (0.97-1.03)	0.103
Bicuspid Aortic Valve	0.42 (0.17-1.02)	0.054
Aortic Root Diameter (continuous)	1.10 (0.92-1.32)	0.309
Ascending Aortic Diameter		
Continuous	1.29 (0.87-1.90)	0.207
≥4 cm	1.96 (0.84-4.58)	0.121
≥4.5 cm	1.47 (0.64-3.37)	0.363
Rheumatic Mitral Valve Disease	1.13 (0.30-4.29)	0.855
Mitral Valve Prolapse/Flail	2.13 (0.97-4.69)	0.159
Mild-to-Moderate Mitral or Tricuspid Regurgitation		
Mitral	3.35 (1.53-7.36)	0.003
Tricuspid	3.62 (1.17-11.23)	0.026
Either	3.02 (1.40-6.50)	0.003
Mitral and Tricuspid Regurgitation Grade (continuous)		
Mitral	5.30 (1.78-15.78)	0.015
Tricuspid	1.69 (0.81-3.55)	0.165
Left Ventricular Ejection Fraction		
Continuous	0.96 (0.93-0.99)	0.011
<50%	2.40 (1.13-5.12)	0.024
Regional Wall Motion Abnormality	1.54 (0.52-4.61)	0.436
Left Ventricular Diastolic Dysfunction		
Any	2.37 (0.28-20.49)	0.432
Grade ≥2	0.73 (0.08-6.40)	0.773
Left Ventricular End-Systolic Diameter (continuous)	1.22 (0.86-1.74)	0.274
Left Ventricular End-Diastolic Diameter (continuous)	1.25 (0.82-1.90)	0.302
Left Atrial Diameter (continuous)	0.96 (0.78-1.17)	0.663
Left Atrial Area (continuous)	1.03 (0.98-1.10)	0.263
Right Ventricular Dysfunction	2.65 (0.60-11.69)	0.198
Right Ventricular Dilatation	2.12 (0.19-24.10)	0.544
Tricuspid Annular Systolic Plane Excursion (continuous)	1.00 (0.99-1.01)	0.993
Pulmonary Arterial Systolic Pressure		
Continuous	1.03 (0.99-1.06)	0.125
>40 mmHg	3.93 (0.92-16.72)	0.164
**Procedural Aspects**		
Urgent Surgery	1.17 (0.23-5.88)	0.851

Biologic (vs Mechanical) Aortic Valve Prosthesis Type	1.58 (0.67-3.73)	0.298
Concomitant Aortic Vascular Intervention		
Any	1.42 (0.64-3.18)	0.392
Composite Graft Use	2.78 (1.16-6.63)	0.022
Concomitant Coronary Artery Bypass Grafting	1.46 (0.60-3.60)	0.406

CI = confidence interval; OR = odds ratio
